# Supplementary material for: Disturbances in Mitochondrial Network, Biogenesis, and Mitochondria-Mediated Inflammatory Responses in Selected Brain Structures of Rats Exposed to Lead (Pb) During Prenatal and Neonatal Development
Source: Int J Mol Sci. 2025 Dec 10;26(24):11907. doi: 10.3390/ijms262411907 (PMC12733045; doi:10.3390/ijms262411907)
Supplement: Supplementary file 1 [file ijms-26-11907-s001.zip › ijms-3964507-supplementary.pdf]

Table S1. The primer pairs used for amplification qRT-PCR.

**Mitochondrial fusion proteins**	
Mfn1	F: ATGGCAGAAACGGTATCTCCA<br>R: GCCCTCAGTAACAAACTCCAGT
Mfn2	F: AGAACTGGACCCAGTTACTA<br>R: CACCTCGCTGATTCCCCTGA
Opa1	F: CCGTGTGAGCAGAAGAACAC<br>R: AGCCTCAAGGCCAACTATGT
**Mitochondrial fission proteins**	
Drp1	F: CAGGAACTGTTACGGTTCCCTA<br>R: CCTGAATTAACTTGTCTCGCGA
Fis1	F: AGAGCACGCAGTTTGAATATGCC<br>R: ATAATCCCGCTGCTCCTCTTT
**Mitochondrial biogenesis proteins**	
Pqargc1	F: TATGGAGTGACATAGAGTGTGCT<br>R: GTCACTACACCACTTCAATCC
Tfam1	F: ATTCCGAAGTGTTTTTCCAGCT<br>R: TCTGAAACTTTTGCATCTGGGT
Nrf1	F: AACACGGAGTGACCCAAAC<br>R: AGTATGTCCGAGTCATCGTAAGA
**Electron transport chain (ETC) complexes**	
mtNd1	F: CTAGCAGAAACAAATCGAGC<br>R: CCTGCGGCGTATTCGACGTT
mtCyb	F: TGCCGAGACGTAAACTACGG<br>R: TAGTCCTCGTCCCACATGGA
mtCo1	F: CCCTGATATAGCATTCCCACG<br>R: ACTGTTCATCCTGTTCCAGC
mtSdha	F: GCTTGCGAGCTGCATTCGG<br>R: TGTGAGCGGGTAGGAAAGAGC
**Interferon-induced genes (ISGs)**	
Mx1	F: CCTCCCACATCTGTAAATCGCTG<br>R: TGTTTTCTTGTGCTTGTACCA
Sting1	F: ACCCCCTTGCAGACCTTGTT<br>R: TTGGTAGACAATGAGGCGGC
Ifi44	F: AACAAGAGGCATTGTTGGGTT<br>R: CGTGTTTGGTGAACCAGGTCT
